# Clinical Implementation of *DPYD* Pharmacogenetic Testing to Prevent Early-Onset Fluoropyrimidine-Related Toxicity in Cancer Patients in Switzerland

**DOI:** 10.3389/fphar.2022.885259

**Published:** 2022-05-18

**Authors:** Ursina B. M. Begré, Markus Jörger, Stefan Aebi, Ursula Amstutz, Carlo R. Largiadèr

**Affiliations:** ^1^ Department of Clinical Chemistry, Inselspital, Bern University Hospital, University of Bern, Bern, Switzerland; ^2^ Department of Medical Oncology and Hematology, Cantonal Hospital St. Gallen, St. Gallen, Switzerland; ^3^ Division of Medical Oncology, Cantonal Hospital Lucerne, Lucerne, Switzerland

**Keywords:** adverse drug reactions, chemotherapy, clinical implementation, dihydropyrimidine dehydrogenase, *DPYD*, early adopter, fluoropyrimidine toxicity, pharmacogenetics

## Abstract

The implementation of pharmacogenetic testing into clinical practice has been a slow process so far. Here, we review the implementation of pre-treatment testing of dihydropyrimidine dehydrogenase gene (*DPYD*) risk variants to prevent early-onset fluoropyrimidine (FP)-related toxicity in cancer patients in Switzerland based on data of a large Swiss diagnostic center. In January 2017, the Swiss Federal Office of Public Health introduced the reimbursement of *DPYD* testing by the compulsory health insurance in Switzerland based on evidence for the clinical relevance of *DPYD*-risk variants and the cost-effectiveness of pre-treatment testing, and on the availability of international guidelines. However, we did not observe a strong increase in *DPYD* testing at our diagnostic center from 2017 to 2019. Only a low number of *DPYD*-testing requests (28–42 per year), concerning mostly retrospective investigations of suspected FP-toxicity, were received. In contrast, we observed a 14-fold increase in *DPYD* testing together with a strong shift from retrospective to pre-treatment test requests upon the release of recommendations for *DPYD* testing prior to FP-treatment in April 2020 by the European Medicines Agency. This increase was mainly driven by three geographic regions of Switzerland, where partner institutions of previous research collaborations regarding FP-related toxicity are located and who acted as early-adopting institutions of *DPYD* testing. Our data suggest the important role of early adopters as accelerators of clinical implementation of pharmacogenetic testing by introducing these policies to their working environment and educating health workers from their own and nearby institutions.

## Introduction

Increasing numbers of clinically relevant gene-drug pairs have been described in recent years resulting in evidence-based pharmacogenetic guidelines. For example, PharmGKB, a public online knowledge base managed by Stanford University, has collected and curated information on over 740 drugs, 175 clinical guidelines and 810 drug label annotations approved by agencies for therapeutic products and pharmaceuticals in Europe (EMA), the United States of America (FDA), Japan (PMDA), Canada (HCSC) and Switzerland [swissmedic; (PharmGKB, 2022; Whirl-Carrillo et al., 2012, 2021)]. Despite the evidence for its clinical relevance, the implementation of pharmacogenetics into clinical practice has not achieved wide uptake because of specific barriers such as common time lags between scientific discoveries and clinical uptake, lack of competence and comfort for use by physicians due to limited education as well as logistical challenges, e.g., timely reporting of genetic test results (Krebs and Milani, 2019; Weitzel et al., 2019; Chang et al., 2021).

The fluoropyrimidines (FP), 5-fluorouracil and its oral pro-drug capecitabine, constitute the backbone of many standard chemotherapy regimens for the treatment of certain solid tumors such as head and neck, gastrointestinal tract, breast and pancreatic cancers (Froehlich et al., 2015; Wigle et al., 2021). However, the occurrence of severe FP-related toxicities in 10%–40% of patients (depending on the treatment regimen) are an important drawback of these drugs, causing severe morbidity or treatment cessation (Froehlich et al., 2015; Amstutz et al., 2018; Henricks et al*.*, 2018). Dihydropyrimidine dehydrogenase (DPD, encoded by *DPYD*) is the critical determinant of systemic 5-FU exposure because the enzyme rapidly inactivates the vast majority of administered 5-FU in the liver (Sommadossi et al., 1982; Desgranges et al., 1986; Heggie et al., 1987; Spector et al., 1993). Thus, reduced activity of DPD is one of the main causes of FP-related toxicity due to the slower degradation of 5-FU resulting in higher exposure of 5-FU and cytotoxic metabolites (Diasio and Harris, 1989; Longley et al., 2003). Patients with reduced DPD activity are at risk of supra-therapeutic drug concentrations if given standard doses and are consequently at risk of developing severe or sometimes even lethal FP-related toxicities. Reduced DPD activity can at least partly be attributed to genetic variability in *DPYD*. Four single nucleotide polymorphisms (SNP) [two missense; c2846A>T and c.1679T>G, and two splice variants; c1129-5923C>G (c.1236G>A/HapB3) and c1905+1G>A] have shown consistent associations with increased 5-FP-toxicity risk and are currently the best clinically validated genetic markers for this risk (Froehlich et al., 2015; Meulendijks et al., 2015; Amstutz et al., 2018).

Various expert groups and medical societies recommend pre-treatment testing of these four genetic variants and published guidelines for clinical practice (Amstutz et al., 2018; Hamzic et al., 2020; Lunenburg et al., 2020; Wörmann et al., 2020). In 2020, the Committee for Medicinal Products for Human Use of the European Medicines Agency (EMA) published a recommendation to screen for DPD deficiency in cancer patients either by *DPYD* genotyping or DPD phenotyping before the use of FP (EMA, 2020; EMA, 2022). To our knowledge, implementation of pre-treatment *DPYD* testing as standard of care has started in several countries, e.g., France, Germany, Canada, with the Netherlands playing a leading role in this process (ANSM, 2018; Lunenburg et al., 2020; Martens et al., 2020; Wörmann et al., 2020; Jolivet et al., 2021). However, a systematic overview on the implementation status is lacking.

In Switzerland, the Swiss Federal Office of Public Health (FOPH) introduced mandatory reimbursement of *DPYD* testing by Swiss health insurers by 1 January 2017 (FOPH, 2017; SSCPT and FOPH, 2016). The FOPH based its decision on the scientific evidence provided in a proposal for reimbursement of pharmacogenetic tests by the Swiss Society for Clinical Chemistry. Therefore, FOPH acknowledged that there was sufficient evidence from a large number of prospective and retrospective studies as well as meta-analyses to warrant *DPYD* testing in all patients prior to FP-therapy initiation (Meulendijks et al., 2015; Deenen et al., 2016).

Here, we review the clinical implementation of *DPYD-* pharmacogenetic testing from the perspective of a large Swiss diagnostic center. More specifically, we assess how different decisions by national and international agencies and publications of guidelines by various stakeholders correlate with the development of test numbers. In addition, we compare expected to observed carrier frequencies and provide data on turnaround time, a crucial variable for pre-treatment testing.

## Diagnostic Center and Data Extraction

The Bern University Hospital has offered *DPYD* testing both to in-house and external clinicians through its diagnostic services, the Center for Laboratory Medicine (ZLM) and the Clinical Genomics Lab (CGL) since 2007. Until 2017, less than ten DYPD tests per year were performed. Since 2017, *DPYD* testing has encompassed specifically the four SNP: c1129-5923C>G (rs75017182) and c2846A>T (rs67376798), c1905+1G>A (rs3918290) and c.1679T>G (rs55886062). Genotyping is carried out using validated allelic discrimination (TaqMan) assays. The tests are performed daily on working days. Interpretation and recommendations in the diagnostic reports are currently based on the guidelines of the Swiss Group of Pharmacogenomics and Personalised Therapy (Hamzic et al., 2020).

For the period from 2017 to 2021, we extracted data from the laboratory information system and from handwritten notes on the order and consent forms for genetic testing (Supplementary Figure S1). For every requested test, the sender’s intention to do either a prospective or retrospective testing as well as the genotype in the result report was recorded. Prospective and retrospective cases were either identified by the respective tick boxes “prospective = before therapy start” and “retrospective = after therapy start”, or clinical information on the order form including the terms “before therapy”, “therapy planned” or a clear statement about a previous toxicity event, respectively.

Turnaround time (TAT) calculations were based on a subset of orders received between 1 July 2020, and 31 July 2021, that had a clear date of blood sampling, recorded on the order form or an additional document sent with the request. We extracted three specific time points for TAT: 1) date and time of the blood draw; 2) date and time when the order had been received by the laboratory; 3) date and time when the test result had been validated and released to the requesting physician. The TAT was defined as time between blood draw and release of results. In addition, we defined the “internal TAT” as time between order receipt and release of result, and the “time to lab” as time between blood draw and receipt of order. Furthermore, for prospective tests including the information about the time of blood draw and planned FP-chemotherapy start, the time between test-result release and planned therapy start was calculated. Finally, geographic locations of requesting institutions were recorded.

## Results

Between 1 January 2017, and 31 December 2021, our laboratory performed a total of 1,048 *DPYD* tests. The majority (57.7%; *n* = 605) was identified as prospective requests, i.e., tests that been ordered with the intention to receive the result prior to the first administration of FP-containing chemotherapy. For the same period, we recorded 13.5% (*n* = 141) retrospective tests (ordered after therapy start) and for 28.8% (*n* = 302) of the tests we could not determine whether they had been ordered before or after FP-therapy.

Overall, 7.0% (CI^95^: 5.5–8.7%; *n* = 73) of investigated samples were from carriers of *DPYD*-risk alleles including two homozygous carriers (c.1129–5923C>G and c.1905+1G>A, respectively) and two compound-heterozygous carriers (c.1129–5923C>G/c.1905+1G>A and c.1905+1G>A/c.2846A>T, respectively). The risk-variant frequency was significantly lower in the prospective group (4.3%; CI^95^: 2.8–6.2%; n_carrier_ = 26) compared to the retrospective group (14.2%; CI^95^: 8.9–21.1%; n_carrier_ = 20). Carrier frequency of the cases with unknown intention to test was intermediate (8.9%; CI^95^: 6.0–12.7%; n_carrier_ = 27), which is suggestive for a mixture of retrospective and prospective cases.

**FIGURE 1:**
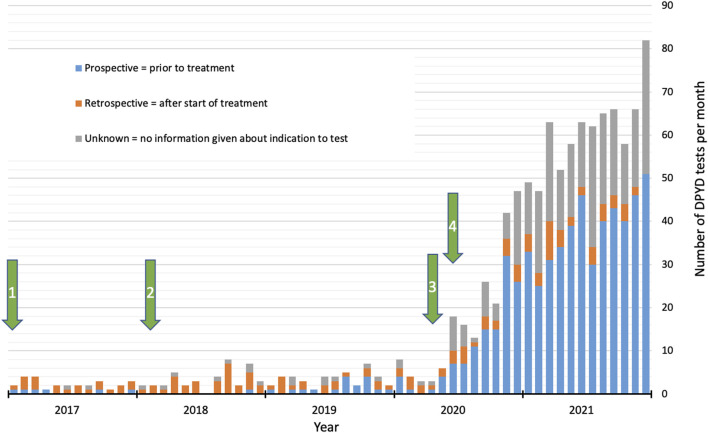
Number of *DPYD* tests per month at the Clinical Genomics Laboratory of Bern University Hospital (Inselspital) from 2017 to 2021, including proportions of intention to test. Arrows indicate the following events: 1) Reimbursement of testing costs by mandatory health insurance; 2) Publication of CPIC guidelines for *DPYD* genotyping; 3) Press release of the EMA recommendations for DPD-deficiency testing; 4) Publication of position paper by the DGHO endorsing EMA recommendations (see text for further details).

A sharp increase in test numbers per month was observed in summer 2020 (Figure 1). This increase coincided with the release of the EMA recommendation for pre-treatment DPD-deficiency testing either by genotyping or phenotyping (30 April 2020) and the publication of a position paper by the German Society for Hematology and Medical Oncology (DGHO) in cooperation with several societies from Austria, Germany, and Switzerland, including the Swiss Society for Medical Oncology endorsing the EMA recommendation [June 15^th^; (Trümper et al., 2020)]. In the period from 1 January 2017 to 30 June 2020, 152 tests were performed (on average 3.6 tests per month) whereas from July 2020 to end of 2021, a total of 896 tests (on average of 49.8 tests per month), which corresponds to 85% of all tests performed from 2017 to 2021 or an approximately 14-fold increase. We also observed a clear shift from retrospective to pre-treatment testing between the two periods (Figure 1; Table 1). This is also reflected by a lower risk-variant carrier frequency (11.2% vs. 6.3%) in the second period (Table 1). As expected from previously published data on *DPYD-*risk variant carriers in Swiss cancer patients (Froehlich et al., 2015), the by far most frequently detected variant was c.1129–5923C>G. Furthermore, the observed carrier frequencies for pre-treatment tests were also in line with previously published data (Table 1).

**TABLE 1:** *DPYD*-risk-variant carrier frequencies and proportions of the intention to test over two time periods: First period lasts from 1 January 2017 to 30 June 2020 and second period from 1 July 2020 to 31 December 2021. Carrier numbers per intention category (prospective = prior to treatment, retrospective = after treatment, unknown = no information given about indication to test) and per variant are shown. Carrier frequencies for Swiss population are from (Froehlich et al., 2015). * indicates the observation of compound heterozygous carriers. ° indicates the observation of homozygous carriers. Note, compound heterozygous carriers are listed twice in the table for each risk allele separately and homozygous carrier only once, which explains that number of carriers given in the totals do not always correspond to the sum of each category.

	*DPYD*-risk variant carriers 01.07.17–30.06.20				*DPYD*-risk variant carriers 01.07.20–31.12.21				Expected carrier frequency in the Swiss population
Intention to test	Prospective *n*	Retrospective *n*	Unknown *n*	Total *n*	Prospective *n*	Retrospective *n*	Unknown *n*	Total *n*	
	%	%	%	%	%	%	%	%	
Number of tests	41	82	29	152	564	59	273	896	
	27.0%	53.9%	19.1%	100%	62.9%	6.6%	30.5%	100%	
c.1129–5923C > G (rs75017182, c.1236G > A/HapB3)	3	4	1	8	18*	5	17°	40*	4.6%
	7.3%	4.9%	3.4%	5.3%	3.2%	8.5%	6.2%	4.5%	
c.2846A > T (rs67376798)	0	1	0	1	3	1*	3	7*	0.6%
		1.2%		0.7%	0.5%	1.7%	1.1%	0.8%	
c.1905+1G > A (rs3918290)	0	6°	1	7	3*	2*	4	9*	0.8%
		7.3%	3.4%	4.6%	0.5%	3.4%	1.5%	1.0%	
c.1679T > G (rs55886062)	0	1	0	1	0	1	1	2	0.4%
		1.2%		0.7%		1.7%	0.4%	0.2%	
Total carrier *n*	3	12	2	17	23	8	25	56	500
%	7.3%	14.6%	6.9%	11.2%	4.1%	13.6%	9.2%	6.3%	6.2%
CI95 [%]	[1.5–19.9]	[7.8–24.2]	[0.9–22.8]	[6.7–17.3]	[2.6–6.1]	[6.0–25.0]	[6.0–13.2]	[4.8–8.0]	[4.3–8.7]

A total of 448 tests was included in our TAT analysis (Supplementary Table S1). The average TAT was 3.1 days (range 0–8 days). For three cases, it took more than 7 days (five working days) to report the result including an average “time to lab” of 2.0 days (range 0–7 days) and an “internal TAT” of 1.1 day (range 0–6 days). A TAT of 7 days is considered to be adequate to avoid therapy delays (Henricks et al., 2018; Hamzic et al., 2020; Varughese et al., 2020; Jolivet et al., 2021). For the 97 requests indicating a planned therapy start, 77.3% of the reports (*n* = 75) were returned to the ordering institution before planned therapy start or on the same day. The result was always reported on time if blood was drawn at least 7 days before planned therapy start.

We received the vast majority of test requests (96.6%) from outside the canton of Bern. The majority (71.9%) of tests were requested by clinicians from Central Switzerland (34.9%), Eastern Switzerland (20.9%) and the canton of Grisons (16.0%), where partner institutions of previous research collaborations regarding FP-related toxicity are located. The remaining requests (28.1%) originated from all other parts of Switzerland. Only 3.4% were requested by clinicians in practices or hospitals in the canton of Bern. We also observed a stronger increase in test requests from the three main sender regions compared to the rest of Switzerland. While these three regions accounted for 51.3% (*n* = 78) of the requests before 1 July 2020, they accounted for 75.3% (*n* = 675) after this date. This trend was even more pronounced when the number of tests was adjusted to the number of inhabitants of different geographic regions (Supplementary Figure S2).

## Discussion

For *DPYD* testing in Switzerland, the acknowledgment of the scientific evidence for clinical benefit by the FOPH and the reimbursement of the test by mandatory health insurance, which are considered to be major hurdles for the clinical implementation of pharmacogenetic tests (Chang et al., 2021; Luzum et al., 2021), have been overcome since January 2017. Furthermore, a guideline to support clinicians to interpret *DPYD* genotypes and to adjust the starting FP dose accordingly had been published by the Clinical Pharmacogenetics Implementation Consortium (CPIC) in 2018 (Amstutz et al., 2018). The availability of guidelines, which are based on a systematic search and review of research findings and provide recommendations for consequences in clinical decision-making, are also considered an important requirement for implementation (Varughese et al., 2020; Chang et al., 2021; Luzum et al., 2021).

However, we did not observe a strong increase in *DPYD* testing at our diagnostic center from 2017 to 2019. Only a low number of patient samples (28–42 per year), concerning mostly retrospective cases of suspected FP-toxicity, were sent for *DPYD* testing. This corresponds to a very small fraction of the approximately 1,000 expected cases receiving FP-based chemotherapy per year in the catchment area of Bern University Hospital (canton of Bern: ca. one million inhabitants in 2020), when taking into account the annual estimate of 1,150 FP-treated patients per million inhabitants for Switzerland (Hamzic et al., 2020). In contrast, we observed a remarkable increase of *DPYD* testing after the release of the EMA recommendation in April 2020. This event led to an active involvement of the prescribers of FP-chemotherapy because the respective medical societies were urged to comment on the EMA statement. Consequently, diverse medical societies including the oncological societies of Austria, Germany, and Switzerland endorsed the EMA recommendation in a consensus paper on existing guidelines for clinical interpretation of *DPYD* genotypes (Wörmann et al., 2020).

These events certainly raised the awareness of oncologists for the clinical benefits of pre-treatment *DPYD* genotyping in order to prevent early-onset fluoropyrimidine-related toxicity, which is reflected by the general shift from retrospective to pre-treatment test requests across Switzerland. However, they cannot fully explain the particular increase in pre-treatment *DPYD* testing in some parts of Switzerland reported here. Within each of these regions, we received most requests from institutions that had previously collaborated with our department in pharmacogenetic studies (Mueller et al., 2013; Gautschi et al., 2015). We hypothesize that these three institutions acted as so-called early adopters of pre-treatment *DPYD* testing in their respective regions because we observed a clustering of newly requesting institutions and small oncology practices, which are located close to the three institutions and collaborate within their region. According to the diffusion of innovations theory, an early adopter in medicine applies new clinical practices before most others, and thus, is important for the implementation of a new practice by providing insights on integration in daily routine and benefits in patient care to their colleagues (Dearing and Cox, 2018). We speculate that the involvement in previous research projects led to the favorable situation turning them into early adopters because they had already been familiar with the concept of pre-treatment *DPYD* testing and had all necessary processes in place, facilitating education of employees and rapid implementation of this new practice. The joint research with these early adopters enabled the optimization of diagnostic processes. This was actually reflected by our TAT analysis showing that *DPYD* testing results can be reliably returned in due time, i.e., without causing treatment delays, which is crucial for sustainable implementation (Henricks et al., 2018; Jolivet et al., 2021).

Another factor that may have played an important role for the widespread clinical implementation in these regions is the high number of patients treated with FPs at these early-adopting centers. Based on the *DPYD*-risk variant frequency of 4.1% observed here (Table 1; pre-treatment group), a relatively large number of approximately 25 patients needs to be screened to observe one actionable genotype on average. Yet, the detection of such risk-variant carriers is essential to visualize the benefit of the screening. An individual physician may treat a substantial number of patients without having a single actionable *DPYD* genotype reported by the laboratory, what may lead to a loss of confidence in the value of the test. Thus, it would be interesting to further monitor the future development of *DPYD*-genotyping requests from small practices.

The question arose to what extent the development of *DPYD* genotyping observed here was representative for the whole country. Laboratories offering pharmacogenetic tests for clinical practice require a permit by the FOPH and are obliged to annually report their activities, including test statistics and the result of external quality control schemes. However, unfortunately, due to data protection reasons we could not access this data. Hence, we conducted a small survey of four Swiss laboratories identified on the internet and two of them provided information. Both laboratories also reported a trend of increasing *DPYD* test numbers in past years (S. Parejo, F. Badiqué, A.-L. Rougemont, pers. commun.). Thus, we assume that the numbers reported here are representative for the general trend of clinical uptake of *DPYD* testing in Switzerland.

In conclusion, our data underline the importance of early adopters for clinical implementation of pharmacogenetic tests. Even when major implementation requirements are fulfilled (i.e., scientific evidence for clinical benefit, availability of guidelines, reimbursement of testing costs, diagnostic work flows facilitating adequate TAT of results, recommendations by official health agencies and their endorsement by the societies of the prescribers), early-adopting clinics—here clinics previously being involved in clinical research on FP-pharmacogenetics—may be pivotal accelerators of clinical implementation by introducing these policies to their working environment and educating health workers from their own and nearby institutions. With regard to future development, these early-adopting regions may be of great importance for successful implementation in other areas of Switzerland, provided that their experience will be shared through active networking among stakeholders, e.g., oncologists, pharmacologists and clinical chemists. Thus, we recommend to identify potential early-adopting clinics in other regions of Switzerland and to actively motivate them to implement pre-treatment *DPYD* testing as the most effective strategy for further establishing this pharmacogenetic test in standard clinical care. Finally, we suggest that identifying early adopters of *DPYD* genotyping in other countries could have a similar impact at an international level.

## Data Availability

The original contributions presented in the study are included in the article/Supplementary Material, further inquiries can be directed to the corresponding author.
