# Supplementary figures and images for: Clinical Implementation of DPYD Pharmacogenetic Testing to Prevent Early-Onset Fluoropyrimidine-Related Toxicity in Cancer Patients in Switzerland
Source: Front Pharmacol. 2022 May 18;13:885259. doi: 10.3389/fphar.2022.885259 (PMC9159275; doi:10.3389/fphar.2022.885259)

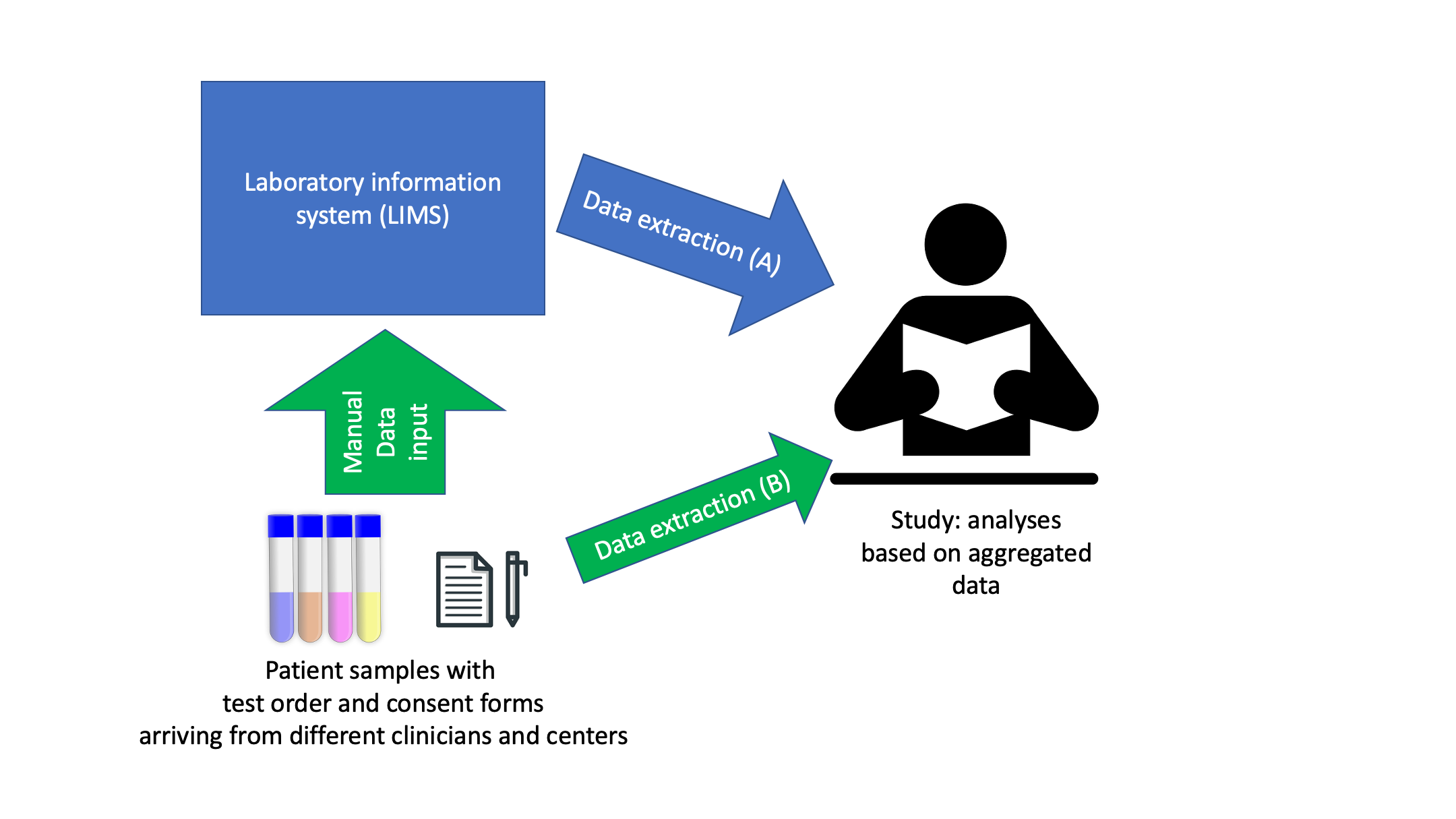

Supplement: Supplementary file 1 [file Image1.tiff]

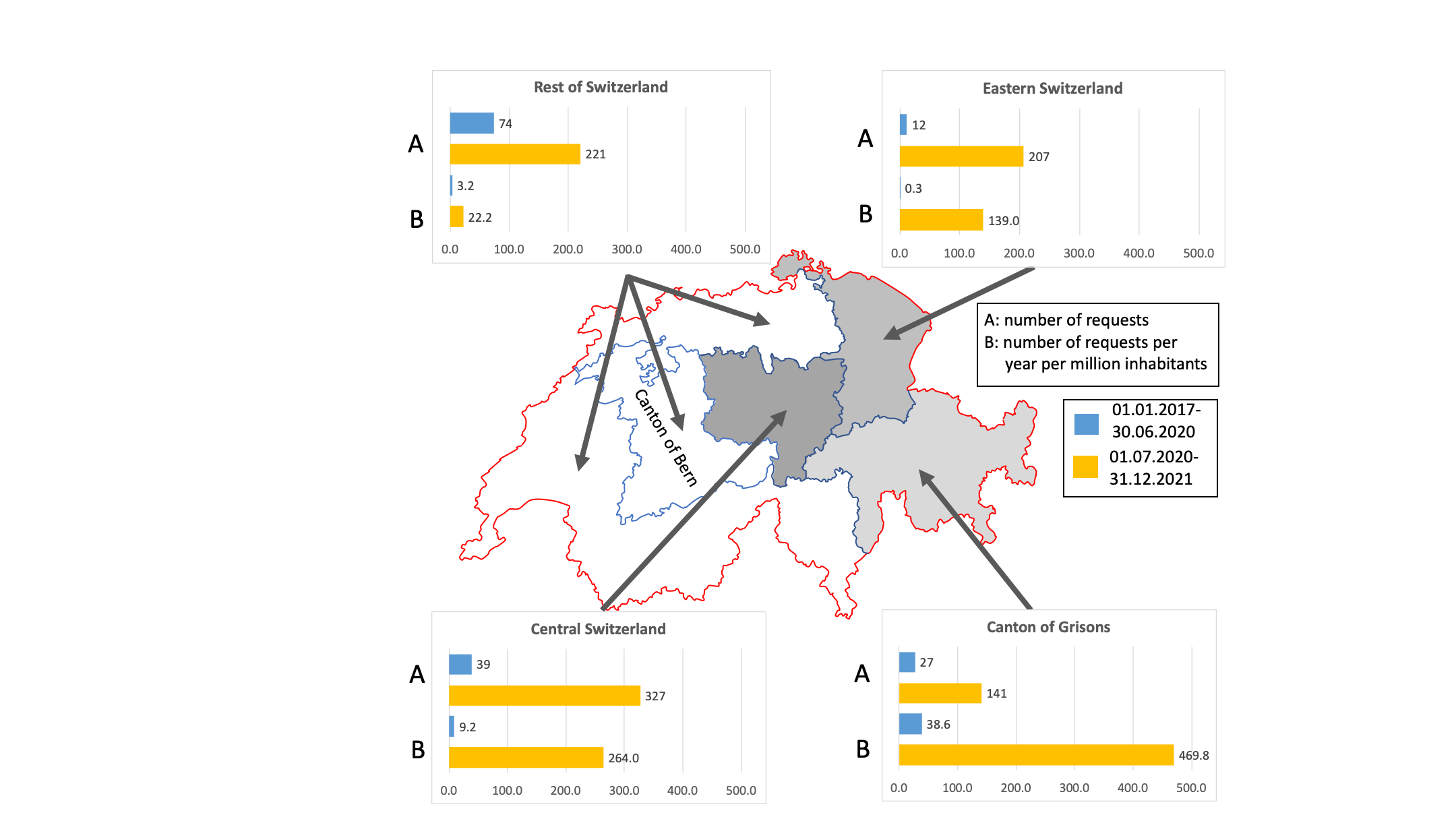

Supplement: Supplementary file 2 [file Image2.tiff]
