# Supplementary material for: Clinical Implementation of DPYD Pharmacogenetic Testing to Prevent Early-Onset Fluoropyrimidine-Related Toxicity in Cancer Patients in Switzerland
Source: Front Pharmacol. 2022 May 18;13:885259. doi: 10.3389/fphar.2022.885259 (PMC9159275; doi:10.3389/fphar.2022.885259)
Supplement: Supplementary file 3 [file DataSheet1.docx]

Supplementary Material

# Supplementary Figures and Tables

## Supplementary Figures

**Supplementary Figure 1.** Flow chart of data collection. (A) Most data regarding genotyping results, turnaround time (TAT) analysis, intention to test, and geographic origin of the requests were extracted directly from the database of laboratory information system (LIMS) of the diagnostic center of the Bern University Hospital (Inselspital, Switzerland). (B) In rare cases, missing information was complemented by reviewing the paper copies of the order and consent forms. See main text for further details.

**Supplementary Figure 2.** Geographic origin of pharmacogenetic *DPYD*-test requests received by the diagnostic center of the Bern University Hospital (Inselspital, Switzerland) from 2017 to 2021. Data displayed on the map of Switzerland are from following geographic regions as discussed in the main text: Central Switzerland, Eastern Switzerland, Canton of Grisons and the remaining parts of Switzerland. Blue bars: requests received from 01.01.2017 to 30.06.2020; yellow bars: requests received from 01.07.2020 to 31.12.2021; Bar charts part A: unadjusted number of requests received from a particular geographic region; Bar charts part B: number of request per year adjusted to the number of inhabitants of the respective geographic region, i.e. number of requests per year per million inhabitants. Source of the Swiss population data for 2020: <https://www.bfs.admin.ch/bfs/de/home/statistiken/bevoelkerung/stand-entwicklung.assetdetail.18344208.html> (Accessed April 14, 2022).

## Supplementary Tables

**Supplementary Table 1.** Turnaround time (TAT) analysis based on 448 pharmacogenetic *DPYD* tests carried out between July 1^st^, 2020, and July 31^st^, 2021 at the diagnostic center of the Bern University Hospital (Inselspital, Switzerland). TAT: time in days from blood draw to release of results; “Time to Lab”: time in days from blood draw to receipt of order in the laboratory; “Internal TAT”: time in days from receipt of order in the laboratory to the release of the test result.

|  | TAT | Time to lab | Internal TAT |
| --- | --- | --- | --- |
| Mean [days] | 3.15 | 2.02 | 1.13 |
| Median [days] | 3.00 | 1.50 | 1.00 |
| SD [days] | 1.57 | 1.33 | 1.14 |
| Range [days] | 0-8 | 0-7 | 0-6 |
